# Nucleolar Expression and Chromosomal Associations in Robertsonian Spermatocytes of *Mus musculus domesticus*

**DOI:** 10.3390/genes10020120

**Published:** 2019-02-06

**Authors:** Fernanda López-Moncada, Daniel Tapia, Nolberto Zuñiga, Eliana Ayarza, Julio López-Fenner, Carlo Alberto Redi, Soledad Berríos

**Affiliations:** 1 Programa Genética Humana, ICBM, Facultad de Medicina, Universidad de Chile, Santiago 8380453, Chile; fernanda.lopez.m@gmail.com (F.L.-M.); danieltapiasalvo@gmail.com (D.T.); nolbertoz@ug.uchile.cl (N.Z.); 2 Departamento de Tecnología Médica, Facultad de Medicina, Universidad de Chile, Santiago 8380453, Chile; elianaayarza@yahoo.com; 3 Departamento de Ingeniería Matemática, Universidad de La Frontera, Temuco, Chile; julio.lopez@ufrontera.cl; 4 Department of Biology and Biotechnology “L. Spallanzani”, University of Pavia, 27100 Pavia, Italy; carloalberto.redi@unipv.it

**Keywords:** nucleoli, NOR, chromosome associations, meiotic prophase, spermatocytes, *Mus m. domesticus*, Robertsonian chromosomes, chromosome translocation

## Abstract

We studied and compared the nucleolar expression or nucleoli from specific bivalents in spermatocytes of the standard *Mus musculus domesticus* 2n = 40, of Robertsonian (Rb) homozygotes 2n = 24 and heterozygotes 2n = 32. We analyzed 200 nuclear microspreads of each specific nucleolar chromosome and spermatocyte karyotype, using FISH to identify specific nucleolar bivalents, immunofluorescence for both fibrillarin of the nucleolus and the synaptonemal complex of the bivalents, and DAPI for heterochromatin. There was nucleolar expression in all the chromosomal conditions studied. By specific nucleolar bivalent, the quantitative relative nucleolar expression was higher in the bivalent 12 than in its derivatives, lower in the bivalent 15 than in its derivatives and higher in the bivalent 16 than its Rb derivatives. In the interactions between non-homologous chromosomal domains, the nucleolar bivalents were preferentially associated through pericentromeric heterochromatin with other bivalents of similar morphology and sometimes with other nucleolar bivalents. We suggest that the nucleolar expression in Rb nucleolar chromosomes is modified as a consequence of different localization of ribosomal genes (NOR) in the Rb chromosomes, its proximity to heterochromatin and its associations with chromosomes of the same morphology.

## 1. Introduction

The nucleolar organizing regions (NORs) are chromosomal segments that contain repeated tandem sequences of ribosomal genes and can produce nucleoli [1,2,3]. In the species *Mus musculus domesticus* (2n = 40) these regions are multiple and are found in the telocentric chromosomes 12, 15, 16, 18 and 19 [4,5]. During the early stages of the meiotic prophase I, the ribosomal genes are actively transcribed, which makes it possible to observe nucleolar material bound to the NOR of the nucleolar bivalent that gives rise to it. The number of nucleoli observed during the meiotic prophase depends on the number of chromosomes with NOR, their transcriptional activity and the associations between nucleolar bivalents that can give rise to common nucleoli [6,7]. All the chromosomes of *M. m. domesticus* present large segments of heterochromatin in sectors adjacent to the centromeres and in the nucleolar chromosomes limiting the NORs [8,9]. Thus, in spermatocytes of *M. m. domesticus* 2n = 40, the suprachromosomal interactions that lead to the formation of nuclear territories would be especially determined by the association of groups of bivalents bound by their pericentromeric heterochromatin and the nucleoli would be part of some of those chromocentres [10]. *Mus m. domesticus* is characterized by natural populations of great heterogeneity in their diploid numbers due to the occurrence of Robertsonian (Rb) chromosome fusions [11,12]. In these chromosomal rearrangements, rupture at the centromere level occurs in two telocentric chromosomes and the subsequent fusion of their long arms, which generates Robertsonian metacentric chromosomes. These rearrangements occur in different combinations between all the telocentric chromosomes [12]. When the Rb fusions involve nucleolar chromosomes, these NORs are structurally preserved because the nucleolar organizing regions are located in the sub-centromeric region of the long arms of the chromosomes in this species [13]. In this way, in the nucleolar chromosomes of Rb mice, the NORs are located close to the centromeric region of a metacentric chromosome and surrounded by the pericentromeric heterochromatin from the two original ancestral telocentric chromosomes [14]. This new chromosomal organization modifies the distribution of NOR and nucleoli in the nucleus and the conformation of the territories in which they participate. In fact, spermatocytes of 2n = 40 mice, which present pericentromeric NORs exclusively in telocentric bivalents, show only nucleoli located in the nuclear periphery, while in Rb homozygous spermatocytes, nucleoli can be observed in the periphery and in the center of the nuclear space [15]. However, it is unknown if the change in the chromosomal position of the NOR (and therefore in the nuclear space) affects the magnitude of its expression in the meiotic prophase [16].

We studied whether the nucleolar expression varies comparing NORs localized in telocentric nucleolar chromosomes with the respective derived Rb chromosomes; as well as the interactions between these and other chromosomal domains in spermatocytes of 2n = 40 mice, homozygous for all telocentric chromosomes; 2n = 24, homozygous for Rb chromosomes; and 2n = 32, heterozygous for Rb chromosomes. 

Nucleolar expression was observed in all the chromosomal conditions studied, being globally higher in the nuclei of spermatocytes 2n = 40 and 2n = 24 than in those from spermatocytes 2n = 32. By specific bivalent, the pattern of nucleolar expression was variable between telocentric nucleolar bivalents and it was modified in the derived Rb nucleolar chromosomes.

## 2. Materials and Methods

### 2.1. Animals

The spermatocytes of six three-months-old *M. m. domesticus* were analyzed. Two were homozygote 2n = 40 CD1 mice with all telocentric chromosomes and five pairs of nucleolar chromosomes numbers 12, 15, 16, 18 and 19. Two mice were Milano II 2n = 24 with three pairs of nucleolar Rb metacentric chromosomes, numbers 10.12, 5.15, 16.17, and two pairs of the telocentric nucleolar chromosomes, 18 and 19. The two heterozygotes Rb 2n = 32 had three single nucleolar Rb metacentric chromosomes, three single nucleolar telocentric chromosomes, numbers 12, 15, 16, and two pairs of telocentric nucleolar chromosomes, numbers 18 and 19. Chromosome numbers are reported according to the 2n = 40 standard karyotype [4,5]. The heterozygote mice were generated by mating females of the laboratory strain CD1 2n = 40 and males of the Milano II race (2n = 24) or the reciprocal crossings. Mice were maintained at 22 °C with a light/dark cycle of 12/12 hours and fed ad libitum. Procedures involving the use of the mice were reviewed and approved by the Ethics Committee of the Faculty of Medicine, Universidad de Chile (Nº CBA #0441) and by the Ethics Committee of the Chilean National Science Foundation FONDECYT-CONICYT.

### 2.2. Karyotyping

Mitotic chromosomes for karyotype were obtained from bone marrow cells using the conventional method, which includes 0.075 M KCl as a hypotonic solution and methanol acetic acid 3:1 v/v as the fixation solution. Metaphase plates were stained with Giemsa (Merck, Darmstadt, Germany) or with 4′, 6-diamidino-2-phenylindole (DAPI).

### 2.3. Spermatocyte Microspreads

Spermatocyte spreads were obtained following the procedure described by Peters et al. [17]. Briefly, testicular cells were suspended in 100 mM sucrose for one minute and then spread onto a slide dipped in 1% paraformaldehyde in distilled water containing 0.15% Triton X-100 then left to dry for two hours in a moist chamber. The slides were subsequently washed with 0.08% Photoflo (Kodak, Rochester, NY, USA), air-dried, and rehydrated in PBS. To identify specific chromosomes, the FISH painting technique was performed and, subsequently, a double IF was performed for the detection of the nucleolar protein fibrillarin and the protein SYCP3, a structural component of the synaptonemal complex (SC).

### 2.4. In-Situ Hybridization (FISH)

Slides containing germ cells prepared as described above were treated for 5 min with PBS, dehydrated in a series of 70, 80, 90, and 100% ethanol for 2 min each and air-dried at room temperature. DNA painting probes specific for nucleolar chromosomes 12, 15 or 16 (Green XCyting Mouse Chromosome Paint Probes, Metasystem) were added to germ cells, covered with coverslips and denatured together at 75 °C for 2 min. Following denaturation, slides were incubated in a humid chamber at 37 °C for 24 h. After incubation, coverslips were removed, and slides rinsed with 0.4 x SSC (saline sodium citrate buffer) at 72 °C for 2 min; 2 x SSC, 0.05% (v/v) Tween20 (Sigma-Aldrich, St. Louis, Louis, MO, USA) at room temperature for 30 s. Finally, cells were rinsed twice in PBS for 5 min each. Heterochromatin was stained with DAPI (4′, 6-diamidino-2-phenylindole) (Calbiochem, San Diego, CA, USA) and coverslips mounted with Vectashield. 

### 2.5. Immunofluorescence (IF)

The slides were incubated for 45 minutes at 37 °C in a moist chamber with the primary antibodies: mouse anti-SYCP3 1:100 (Santa Cruz Biotechnology, ab74569) and rabbit anti-Fibrillarin 1:100 (Abcam, Cambridge, UK, ab5821). Then, the slides were incubated for 30 min at room temperature with the secondary antibodies: FITC-conjugated goat anti-mouse IgG (1:50) (Sigma), or Texas red-conjugated goat anti rabbit IgG (1:200) (Jackson ImmunoResearch). Slides were counterstained with 1 μg/mL DAPI (4′, 6-diamidino-2-phenylindole). Finally, slides were rinsed in PBS and mounted in Vectashield (Vector Laboratories).

### 2.6. Images Analysis

Photographs were obtained using a Nikon (Tokyo, Japan) Optiphot microscope equipped with epifluorescence optics, using the same parameters of exposition and intensity for each picture. Images were analyzed using Adobe Photoshop CS5.1 software (San José, CA, USA) and the Magic Wand Tool to delineate the area of fibrillarin defined by the intensity of red color. The nucleolar expression was established measuring the pixels (px) of the fibrillarin areas. Then, to collectively visualize the bivalents, fibrillarin and heterochromatin, a fusion of the layers was performed using the Lighten tool. 

### 2.7. Statistical Analysis

A comparison of proportions was made with the Z score test using the ratio difference estimator (δ). The normality of the sample distribution in the variables to be tested was determined using the Kolmogorov Smirnov test. For variables without normal distribution, the comparison of means was made with the nonparametric Kruskal–Wallis test. The confidence interval used for all tests was 95% with a level of significance of 5% (α < 0.05). All calculations were performed in the Analyze-it^®^ statistical software used in Microsoft Excel^®^ 2010.

## 3. Results

### 3.1. FISH and IF in the Detection of Nucleolar Expression in Specific Bivalents of Mouse Spermatocytes

**Figure 1:**
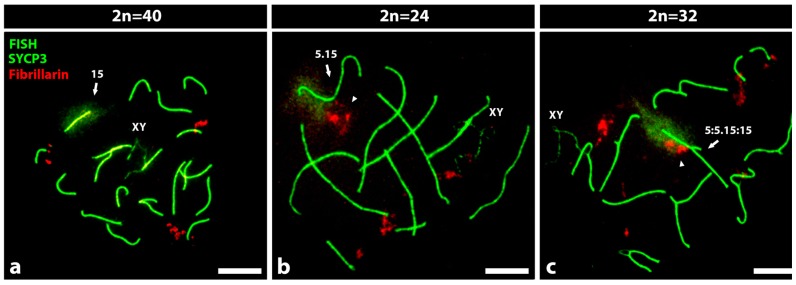
Nuclear microspreads of spermatocytes 2n = 40, 2n = 24 and 2n = 32. By immunocytochemistry, the synaptonemal complex (SCs) of the bivalents (green) and the fibrillarin of the nucleoli (red) are shown. By fluorescent in situ hybridization (FISH) and a specific probe, the chromatin of the nucleolar bivalent 15 (a), or the Rb nucleolar bivalent 5.15 (b) and the trivalent 5:5.15:15 (c), were identified (green). Arrowheads show the nucleoli associated with these nucleolar chromosomes. XY = sex bivalent. Bar = 10 µm. (**a**) The bivalent 15 is indicated among the SCs of nineteen telocentric bivalents, three of them with associated nucleoli. The bivalent 15 does not present associated nucleolus. (**b**) The bivalent 5.15 is indicated among the SCs of 8 metacentric autosomal bivalents, 3 telocentric autosomal bivalents and the sex chromosome pair (XY). The arrowhead indicates the nucleolus associated with the middle of the Rb nucleolar bivalent 5.15. (**c**) The trivalent 5:5.15:15 is indicated among 8 trivalents, 3 telocentric autosomal bivalents and the sex chromosome pair (XY). The arrowhead indicates the nucleolus associated with the middle of the trivalent 5:5.15:15.

As example of our findings, we show (Figure 1) three representative images of nuclear microspreads of spermatocytes 2n = 40, 2n = 24 and 2n = 32, in which the SCs and the fibrillarin were detected by immunofluorescence and chromosome 15 by FISH. In nuclear microspreads of spermatocytes 2n = 40, it is possible to detect the SCs of the 19 autosomal bivalents and the partial synapses between the sex chromosomes X and Y. The nucleolar bivalent 15, revealed by the green chromatin, showed a non-associated nucleolus (Figure 1a). In spermatocytes 2n = 24, the SCs of 8 metacentric autosomal bivalents, 3 telocentric autosomal bivalents, in addition to the sexual pair in partial synapses, were observed. The Rb nucleolar bivalent 5.15, revealed by the green chromatin in one of its arms, also presented one associated nucleolus (Figure 1b). Finally, in the 2n = 32 spermatocytes, the SCs of 8 autosomal trivalents, 3 autosomal telocentric bivalents and the sexual pair XY in partial synapses were observed. The Rb nucleolar trivalent 5:5.15:15, revealed by the green chromatin in one of its arms, also presented one associated nucleolus (Figure 1c). The trivalents presented a complete synapse between the long arms of the Rb metacentric and the telocentric chromosomes, while the short arms of the latter ones could be unsynapsed or partially synapsed.

To establish the global nucleolar expression between the spermatocytes with different chromosomal constitutions, the pixels (px) of the fibrillarin zones per each nucleus defined by intensity of red color were quantified using the Adobe Photoshop CS5 image software.

The nucleolar expression was higher in 2n = 40 and 2n = 24 spermatocyte nuclei in comparison to 2n = 32 spermatocytes. On average, 4241 px of fibrillarin was estimated in spermatocyte nucleus 2n = 40, 4201 px in 2n = 24 and 3161 px in 2n = 32. The differences between the means of total nucleolar expression of 2n = 40 and 2n = 32 and between 2n = 24 and 2n = 32 were statistically significant (*p* < 0.001). The smallest nucleolar bivalents 18 and 19, present in all the spermatocyte’s karyotypes, always expressed large and independent nucleoli, thus constituting a good positive control of nucleolar expression. The FISH method subjects the cells (spermatocytes) to very high temperatures that denature proteins, so their subsequent detection by immunofluorescence is not always possible. To evaluate this negative effect on fibrillarin, 100 nuclear microspreads of spermatocytes 2n = 24, 50 of them treated with FISH and 50 without FISH, were evaluated (not shown). An average of 878 px of nucleolar area associated with nucleolar bivalent 19 was obtained without FISH, whereas an average of 1136 px was obtained with FISH. Between these values, no significant difference was observed (*p* = 0.0631). 

**Figure 2:**
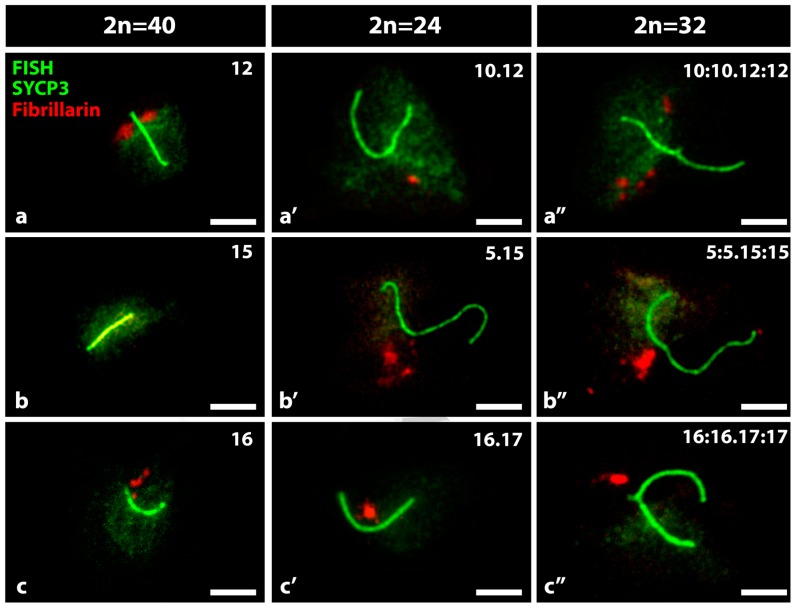
Nucleolar expression in specific ancestral nucleolar bivalents in spermatocytes 2n = 40 and their nucleolar derivatives in spermatocytes 2n = 24 or 2n = 32. (**a**) Bivalent 12; (**a′**) bivalent Rb 10.12; (**a″**) trivalent 10:10.12:12; (**b**) Bivalent 15; (**b′**) bivalent Rb 5.15; (**b″**) trivalent 5:5.15:15; (**c**) Bivalent 16; (**c′**) bivalent 16.17; (**c″**) trivalent 16:16.17:17. By immunocytochemistry, the SCs of the bivalents (green) and the fibrillarin of the nucleoli (red) are shown. By FISH and a specific probe, the chromatin of the nucleolar bivalents, or derivatives, were identified (Green). Bar = 10 µm.

A total of 1800 nuclear microspreads were analyzed, 200 nuclear microspreads for each specific nucleolar chromosome and in the three conditions of spermatocyte’s karyotypes. No differences were found between spermatocytes from animals with the same chromosomal condition. The specific nucleolar bivalents were identified by FISH staining and by immunofluorescence of the proteins fibrillarin and SYCP3, respectively, to recognize nucleoli and the SCs of the bivalents (Figure 2). We compared the nucleoli produced by the telocentric nucleolar bivalents 12, 15 and 16 present in 2n = 40 spermatocytes, with those of the respective Rb nucleolar bivalents, Rb 10.12, 5.15 and 16.17 in 2n = 24 spermatocytes, and with those of the respective nucleolar trivalents in 2n = 32 mice (Figure 2). In all cases, multiple nucleoli were observed for each nucleus although the magnitude of the fibrillarin area was variable and with a heterogeneous distribution of intensities. In the telocentric nucleolar bivalent the fibrillarin was observed adjacent to one or both sides of one of the ends of the SC. In the metacentric Rb nucleolar bivalents, fibrillarin was located toward the middle of the SC, whose length was approximately twice that of the telocentric bivalents. In the nucleolar trivalents, the fibrillarin label was located close to the meeting zone of the centromere of the three chromosomes. In spermatocytes 2n = 40, 100% of the nucleolar bivalents 12 had large nucleoli greater than 3 μm in average diameter; only 35% of the bivalents 15 had nucleoli which were small in size, smaller than 1 μm in diameter, and 90% of the nucleolus bivalents 16 had nucleoli of medium size of 2 μm in average diameter (Figure 2a–c). Seventy three percent of the nucleolar bivalents Rb 10.12 and 90% of the nucleolar bivalents Rb 5.15 and 16.17 had medium-sized nucleoli (Figure 2a′–c′). Ninety percent of the trivalents 10:10.12:12 and 5:5.15:15, and 76% of the trivalents 16:16.17:17 had medium nucleoli (Figure 2a″–c″). 

**Figure 3:**
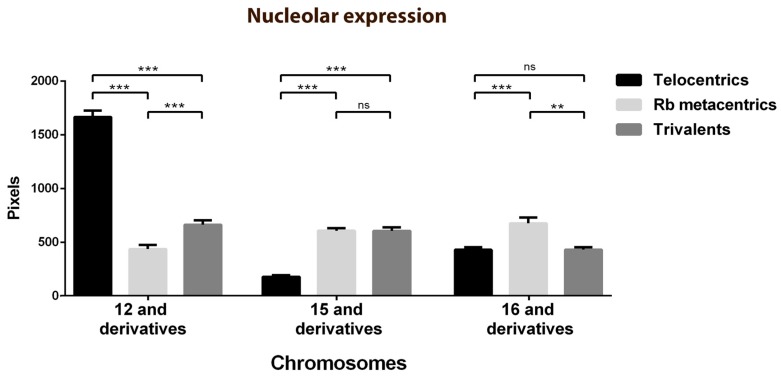
Nucleolar sizes associated with the telocentric bivalents 12, 15 and 16 and their derived chromosomes estimated by the pixels corresponding to the fibrillarin areas. The expression of NORs of bivalents 12, 15 and 16 (black bars) was variable and much higher in the bivalents 12, although the differences were statistically significant among the three. The nucleolar expression between the Rb nucleolar bivalents (light bars) or between the nucleolar trivalents (grey bars) showed no significant differences. The nucleolar expression of bivalent 12 was statistically higher than its derivatives, in bivalent 15 it was statistically lower than its derivatives and in bivalent 16 it was statistically lower than its Rb derivatives and showed no significant differences with the trivalents. Data are expressed as mean ± SEM: ns: *p* > 0.05, **: *p* ≤ 0.01, ***: *p* ≤ 0.001 (Kruskal–Wallis test). Rb: Robertsonian.

To estimate the nucleolar sizes, the pixels corresponding to the fibrillarin areas of the telocentric bivalents 12, 15 and 16 were quantified and compared with those of the derived chromosomes (Figure 3). The estimated nucleolar expression showed statistically significant differences between bivalent 12 and its derivatives, between bivalent 15 and its derivatives, and between bivalent 16 and only its Rb derivatives. The expression of the NORs of the telocentric bivalents 12, 15 and 16 was variable and much higher in the bivalents 12, although the differences were statistically significant among the three. It is also important to take into account that only 35% of the nucleolus bivalents 15 presented a nucleolus. When comparing the nucleolar expression between the Rb nucleolar bivalents, no significant differences were found, as well as comparing the nucleolar expression between the nucleolar trivalents.

### 3.2. Bivalent Associations from Different Shaped Nucleolar Chromosomes Present in Spermatocytes of Mus m. domesticus 2n = 40, 2n = 24 and 2n = 32

**Figure 4:**
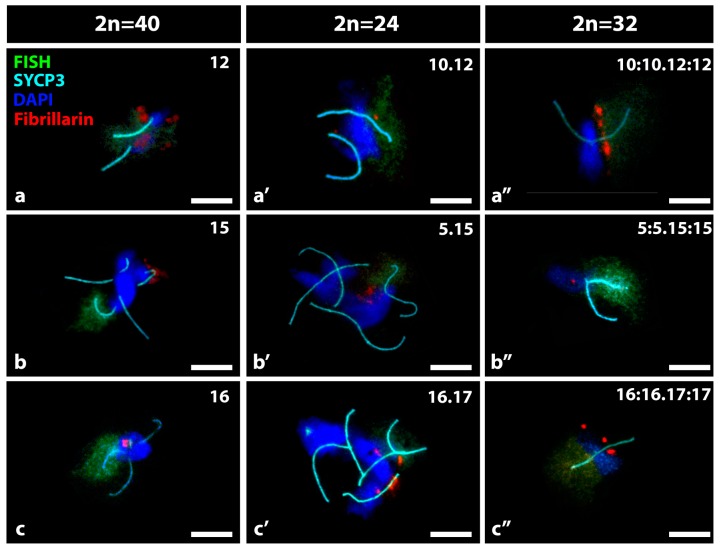
Bivalent associations where a specific nucleolar bivalent is forming part, in spermatocytes 2n = 40 and nucleolar derivatives, in spermatocytes 2n = 24 or 2n = 32. (**a**) bivalent 12; (**a′**) bivalent Rb 10.12; (**a″**) trivalent 10:10.12:12; (**b**) bivalent 15; (**b′**) bivalent Rb 5.15; (**b″**) trivalent 5:5.15:15; (**c**) bivalent 16; (**c′**) bivalent Rb 16.17; (**c″**) trivalent 16:16.17:17. By immunofluorescence the SCs of the bivalents (light blue) and the fibrillarin of the nucleoli (red) are shown. The specific nucleolar bivalents/trivalents were identified by FISH with a probe against the chromatin of the respective nucleolar telocentric chromosome (green). The heterochromatin was stained with DAPI (blue). Bar = 10 µm. (**a**) Bivalent 12 is producing an abundant nucleolus and it is associated through heterochromatin to another bivalent; (**b**) bivalent 15 is not producing a nucleolus and it is associated through heterochromatin to three other bivalents one of them a nucleolar one that has a nucleolus associated; (**c**) bivalent 16 is producing a nucleolus and is associated through heterochromatin to three other bivalents; (**a′**) bivalent 10.12 is producing a small nucleolus and it is associated through heterochromatin to another metacentric bivalent; (**b′**) bivalent 5.15 is producing a medium nucleolus and it is associated through heterochromatin to three metacentric bivalents; (**c′**) bivalent 16.17 is not producing a nucleolus and it is associated through heterochromatin to three metacentric bivalents; (**a″**); (**b″**) and (**c″**) all show one nucleolar trivalent with a nucleolus and not associated with other bivalents at all.

The configuration of associations between non-homologous chromosomes in which the specific nucleolar bivalents were involved was studied in spermatocytes 2n = 40, 2n = 24 and 2n = 32. In addition to the specific nucleolar bivalent, the number of SC from other bivalents, the heterochromatin of the associated chromosomes and the presence of other nucleoli were considered (Figure 4). The nucleolar bivalents 12, 15 and 16 had a similar behavior, remaining alone (15%) or in association through heterochromatin with between 2 and 5 bivalents (70%). In one third of these associations there was an additional nucleolus (Figure 4a–c). The Rb nucleolar bivalents were associated through heterochromatin with other metacentric Rb at different approximate frequencies: Rb10.12: 68% associated between 2 and 8 and 32% not associated (Figure 4a′); Rb5.15: 85% associated between 2 and 8 and 15% non-associated (Figure 4b′); Rb16.17: 90% associated between 2 and 8 and 10% non-associated (Figure 4c′). The additional nucleoli were more frequent when increasing the associated bivalents. Nucleolar trivalents were generally isolated (50–70%) or in associations of only two (20–40%) (Figure 4a″–c″). The nucleolar bivalents do not seem to associate preferentially with other nucleolar bivalents and when they participate in the same cluster of heterochromatin the nucleoli are not linked together.

**Figure 5:**
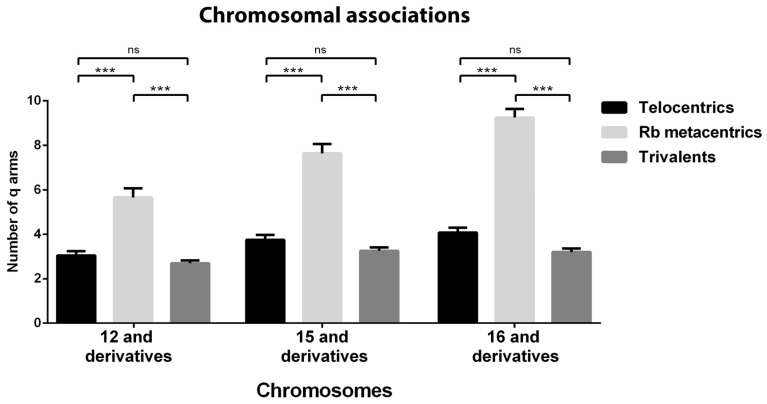
Number of chromosomal arms committed in associations in which a specific nucleolar bivalent is forming part, in spermatocytes 2n = 40 and nucleolar derivatives in spermatocytes 2n = 24 or 2n = 32. In 2n = 40 spermatocytes: an average of 4 chromosome arms were present in chromosomal associations in which NORs of bivalents 12, 15 and 16 were involved (black bars), without significant differences among the three situations. In 2n = 24 spermatocytes: between 6 and 10 chromosome arms were present in chromosomal associations in which NORs of Rb nucleolar bivalents were present (light bars). There were significant differences among the three groups of chromosomal associations. In 2n = 32 spermatocytes: an average of 3 chromosome arms were present in chromosomal associations in which NORs of trivalents were involved (grey bars), without significant differences among the three groups of chromosomal associations. The number of chromosome arms in chromosomal associations defined by a Rb nucleolar bivalent is about twice that observed in the chromosomal associations defined by a telocentric nucleolar bivalent or a nucleolar trivalent. Data are expressed as mean ± SEM ns = *p* > 0.05, *** = *p* ≤ 0.001 (Kruskal–Wallis test).

Taking into account that chromosomes of very different lengths are involved in these associations in which at least one nucleolar bivalent was present, we quantified the number of chromosomal arms involved and the results were expressed in a bar graphic (Figure 5). It was observed that the maximum number of chromosomal arms was concentrated in the associations involving Rb nucleolar bivalents and the minimum was concentrated in the territories defined by a nucleolar trivalent (Figure 5). The association of 4 or 5 Rb metacentric bivalents equals the association of 8 or 10 ancestral telocentric bivalents, a value that is much higher than the average of 3 in each cluster of associated bivalents in spermatocyte 2n = 40. Nucleolar trivalents remained isolated in a manner similar to that observed in non-nucleolar trivalents. Each one of the non-associated trivalents compromised a number of chromosomal arms without significant differences in the respective ancestral telocentric bivalents (Figure 5).

Through pericentromeric heterochromatin, bivalents of the same morphology associate, whether telocentric or metacentric. In all of these associations, one or more nucleoli surrounded by heterochromatin were observed. Depending on whether the chromosome associations are constituted by telocentric or metacentric bivalents, it would be expected they were distributed respectively in the periphery or in the center of the nuclear space.

## 4. Discussion

Metacentric Rb chromosomes can become numerous in the *Mus* genome leading to a reduction of ancestral telocentric chromosomes and to an emergence of new mixed karyotypes [11,12,18]. Furthermore, crossing between wild homozygotes 2n = 40 and Rb homozygotes produce F1 hybrids in whose genomes the ancestral telocentric chromosomes are reunited with the metacentric derivatives [19]. During the meiotic prophase I, trivalents with a Rb metacentric chromosome synapsed with two telocentric chromosomes are formed [20,21]. In this work we try to elucidate how or how much gene expression is affected in these new chromosomal and nuclear conditions approaching it through the analysis of NOR expression that produces nucleoli in spermatocytes with different chromosome constitutions. In somatic cells, it is becoming increasingly evident that chromatin organization within the three-dimensional nuclear space (in other words the genome architecture) is itself a likely factor affecting gene regulation and the systemic control of expression of multiple gene loci [22,23,24]. A similar relationship of the structure and function of the nucleolus has been proposed, whose transcriptional changes would be dependent on the changes in the associated chromatin [25,26]. However, little is known about the organization of nucleolar chromatin in meiotic cells and how this can affect their expression.

The simultaneous application of two methods of difficult compatibility, such as FISH and immunofluorescence (IF), was carried out as has been previously described [27]. Both methods allowed us to identify in each analyzed spermatocyte, a specific nucleolar bivalent and its associated active nucleoli through the identification of fibrillarin protein which is a key factor in nucleolar architecture serving essential functions in rRNA maturation [28]. At the same time, the use of IF for the SYCP3 protein constitutive of the synaptonemal complex, and the use of DAPI, allowed us to observe all the bivalents and if they were associated with each other through the pericentromeric heterochromatin (DAPI). 

In 2n = 40 mice, the nucleolar organizing regions (NOR), where the ribosomal genes concentrate, are located in the sub-centromeric region of the long arms of 5 pairs of telocentric chromosomes [4,5]. Therefore, when the Rb fusions involve nucleolar chromosomes, the NORs are structurally preserved [14] unlike the Rb human chromosomes that usually lose the ribosomal genes [29]. Consequently, in the three Rb nucleolar chromosomes of 2n = 24 mice, NORs are located close to the centromeric region and surrounded by the pericentromeric heterochromatin coming from the two original ancestral telocentric chromosomes [14,15]. However, little is known on whether the change of the NOR chromosomal position, and therefore its interaction with other bivalents, affects the magnitude of ribosomal expression in the meiotic prophase. Mice with the 2n = 40 all telocentric karyotype and those carriers of Rb metacentric derived chromosomes are an unbeatable material in the approach to this comparative analysis of nucleolar expression. We observed nucleoli in all the chromosomal conditions studied, and that the observed variability in nucleolar expression of NORs located in the ancestral nucleolar bivalents 12, 15 and 16, was lost in the derived chromosomes. Quantitatively, the nucleolar expression was decreased in the NORs located in the chromosomes derived from 12, increased in the derivatives of 15, and slightly higher in the chromosomes derived from the nucleolar bivalent 16. In the respective derived Rb nucleolar bivalents, as well as in the nucleolar trivalents, the expression of NORs becomes relatively flat. This expression profile could be a consequence of the new environment where NORs are immersed in a greater amount of pericentromeric heterochromatin and in a different disposition with respect to the transcriptional machinery in the nuclear space [10,30]. In most eukaryotes, NORs have an evolutionary conserved positioning in the chromosomes, generally surrounded by constitutive heterochromatin. It has been proposed that such heterochromatin, instead of silencing NORs transcriptionally, may regulate important unknown features of nucleolus formation [31,32]. These, or other suggested mechanisms, that regulate transcription of ribosomal genes, could be affected by the new location of NORs, considering the higher amount of heterochromatin and the apparent different qualities of them [32]. 

During prophase I of meiosis the SC’ organization and trajectory determine the chromosomal domain topology within the nuclear space [33,34,35,36]. Considering additionally that the nucleolus persists, bound to the NOR that it originates from [37], interactions or associations among heterologous NORs will depend on the real possibility of establishing contacts between them, particularly in the species with multiple nucleolar chromosomes where these options could be present [10].

Thus, in 2n = 40 mice, the NORs of 5 nucleolar bivalents may interact in the peripheral nuclear space, while in 2n = 24 mice, the NORs of the two nucleolar telocentric bivalents can only interact at the nuclear periphery and the NORs of the three Rb nucleolar bivalents toward the nuclear center. As we have shown here in the spermatocytes 2n = 24, bivalents from these two groups do not interact with each other. With the increase of the Rb fusions in meiotic cells, the real possibilities of interactions or free associations between heterologous chromosomal domains are progressively restricting what can favor or channel the chromosomal or genomic evolution of that karyotype towards a certain path.

We define a nuclear territory in the spermatocytes as a place of the cell nucleus in which topological and functional relationships (or chromosomal associations) are established between bivalents or non-homologous chromosomal domains, which generally originate in the early prophase, frequently mediated by constitutive heterochromatin and can be relatively stable throughout meiotic prophase I [10]. In this work, we selected those chromosomal associations in which specific nucleolar bivalents were involved in order to evaluate whether their morphology or nucleolar expression were gravitating factors in the quality or number of the elements that made up the whole of that chromosomal association.

We did not find a relationship between the magnitude of the nucleolar expression and the number of associated bivalents. Neither was there a preferential aggregation between NORs of different nucleolar bivalents as has been proposed for nucleolar chromosomes considering their structural and functional affinities that could favor the consolidation of such interactions [38,39]. 

The nucleolar associations were clearly mediated by constitutive pericentromeric heterochromatin and between bivalents of the same morphology, not preferentially between nucleolar bivalents. In other words, the chromosomal associations were conformed by telocentric bivalents or by metacentric bivalents. The associations of 4 or 5 Rb metacentric bivalents equal the association of 8 or 10 ancestral telocentric bivalents, a value that at that same describes the subtracted telocentric chromosomes from the possibilities of associations among them. Focusing on the perspective of the associable bivalents or those that still have possibilities of interaction with others in the nuclear periphery, this is a remarkable change of the original nuclear architecture and that would explain how the new chromosome path is oriented towards the metacentry [40,41].

Trivalents were generally observed not associated with each other or with other bivalents. They and their isolated behavior are an example of the occupation of the same space, in this case 8 trivalents available at the nuclear periphery does not necessarily lead to an association between them. Neither does the presence of abundant heterochromatin by itself or that of active NORs because at least three of the 8 trivalents share all these characteristics and still persist isolated. There is still a lot to understand with respect to what conditions finally make a chromosomal association become consolidated.

It seems clear that chromosomal changes that have already experienced full establishment in a population or reproductive community at the cellular level mean that a huge transformation of the chromosomal associations where they were forming part, and consequently it would be expected they also change the original nuclear architecture. In this sense, Rb nucleolar chromosomes of homozygous mice possibly have been successful since they are multiple and have reached new chromosomal interactions constituting different chromosomal associations, which include new patterns for nucleolar expression.
